# What engagement strategies are useful in facilitating the implementation of electronic health records in health care settings? A rapid review of qualitative evidence synthesis using the normalization process theory

**DOI:** 10.1177/20552076241291286

**Published:** 2024-11-03

**Authors:** Campion Zharima, Samantha Mhlanga, Saira Abdulla, Jane Goudge, Frances Griffiths

**Affiliations:** 1 Centre for Health Policy (CHP), Faculty of Health Sciences, University of Witwatersrand, Johannesburg, South Africa; 2 Medical School, University of Warwick, Warwick, UK

**Keywords:** User engagement, electronic health records, implementation, qualitative, healthcare setting, hormalization process theory, rapid review

## Abstract

**Objective:**

The study aimed to identify and describe the engagement strategies used in implementing electronic health records in health care settings and to ascertain why they were successful or not, using normalization process theory.

**Methods:**

In this rapid review, we searched PubMed and CINAHL for qualitative and mixed methods primary studies published from 2010 to 2023 (June). We identified 41 studies that explored the implementation of EHRs, involving clinicians as participants. For quality appraisal, we employed the standards for reporting qualitative research (SRQR) tool. For analysis, a qualitative comparative analysis, using the normalization process theory was conducted. This was followed by a narrative synthesis to compile and analyze key findings.

**Results:**

About 56% (*n* = 23) of the studies were conducted in hospitals, while the remaining were done in mental health, maternity, and ambulatory care settings. Participants included a range of clinicians such as nurses, physicians, doctors, dentists, pediatricians and other specialists. Evidence shows that prior to implementation, effective communication of the vision of EHRs and early user involvement in decision-making are useful engagement strategies in preparing users for implementation. Tailored training and on-demand technical support for users sustain system usage during the roll out. Lastly, ongoing engagement with users is essential for continuous user support and system improvements.

**Conclusion:**

User engagement improves the chances of successful implementation, particularly if engagement strategies are effective for the specific stages of implementation. The success of these strategies is more evident when they ensure normalization process theory tenets, which include user coherence, cognitive participation, collective action and reflective monitoring.

## Introduction

Electronic health records (EHRs) are a patient-centric information resource for clinicians and a longitudinal record of patient information and service provision for managers.^1,2^ They enable clinicians to perform their duties more efficiently by improving diagnostic precision, coordination and delivery of care and clinical decision making,^2–4^ thereby acting as a tool for recording, handling and sharing health data. The World Health Organization (WHO) urges governments to invest in digital health solutions like EHRs, especially for low and middle income countries working towards universal health coverage.^5,6^

Despite the potential for EHRs to improve the delivery of health care, implementing EHRs is often difficult and can result in negative and unanticipated disruptions to clinician workflow.^7^ Organization leaders exert greater control over the implementation processes, while end users often have a more intimate understanding of the care delivery process.^8^ Where leaders fail to meaningfully engage end users, it leads to implementation failures.^9^Health care settings are dynamic networks of interactions between people and processes.^10^ Introducing a new sub-system such as an EHR in health care settings has implications for end users and their workflow patterns.^11^ As a result, implementing EHRs requires a synergistic approach involving a top-down and bottom-up direction, with the latter gained through user engagement.

User engagement can include activities to consult, involve or partner with end users of EHRs throughout the implementation process, thereby presenting opportunities to identify and solve implementation challenges in real time.^9,12,13^ Activities could be involving users in decision making processes, partnering with them to co-create solutions, conducting focus groups or feedback sessions to understand user needs. Engagement ensures that implementation is a collaborative process, ensuring that end users’ needs and workflows are accommodated, to promote ownership and acceptance of the new system.^11^

### Evidence on the implementation of EHRs

Evidence on the implementation of e-health systems, including EHRs, has been synthesized in two notable reviews, both of which utilized theoretical frameworks: the Consolidation Framework for Implementation Research (CFIR)^7^ and the normalization process theory (NPT).^14^ The CFIR focuses on the contextual determinants of implementation outcomes while the NPT explains the behaviors and thoughts underlying technology adoption by users.^15^ Although these two reviews are not specific to EHRs, they elaborate on the importance of user engagement and deploy useful theoretical frameworks.

The Ross review, using the CFIR shows the importance of stakeholder engagement during the development, planning and execution of implementation processes.^7^ The Mair review, using the NPT concluded that user engagement is central to successful implementation of new technology, but there are limited efforts aimed at actively involving health professionals during implementation.^14^ Although both reviews underscore the importance of user engagement, they do not explore engagement strategies and factors that promote or inhibit user engagement. While the CFIR carries a wider focus on broader factors of implementation, the NPT carries more depth in explaining the mechanisms of user engagement.^7^

There are several reviews on EHR implementation which report on the factors affecting implementation such as the influence of organizational, human and technological factors.^16–18^ While these reviews emphasize implementation strategies including user involvement, they fall short of synthesizing the strategies necessary for effective user engagement. In this rapid review, we identify and describe the engagement strategies used in implementing electronic health records in health care settings, whether they were perceived as successful and, using NPT, to ascertain the reasons why they were successful or not.

### Normalization process theory (NPT)

We use NPT for this review, a framework developed to fill the gap in addressing the work of individuals and groups during implementation.^14,19^ The NPT explores the factors influencing the routine embedding of complex interventions into everyday practice.^20^ Thus, the theory holds practical value by offering a means to assess implementation from a user's perspective.^21,22^

The theory incorporates four constructs, used in this review to evaluate engagement strategies: coherence (or sense-making), cognitive participation, collective action and reflexive monitoring.^20,23^ These constructs interact and influence one another. Table 1 below shows the definitions of the NPT's constructs and the operational definitions used for this review.

**Table 1.** Applied NPT constructs as defined by Finch et al.^23^

NPT construct	Conceptual definition by Finch et al.	Operational definition for engagement strategies
Coherence or Sense Making	The process of sense-making and understanding that individuals and organizations have to go through in order to promote or inhibit the routine embedding of a practice to its users. These processes are investments of *meaning* made by participants.	Does the strategy enable users to understand and make sense of the new system, how it differs from the old, the value of the new practice and the work that needs to be done to integrate the new system into their workflow?
Cognitive Participation	The process that individuals and organizations have to go through in order to enroll other individuals to engage with the new practice. These processes are investments of *commitment* made by participants	Does the strategy include key personnel, build user relationships for support, drive change, establish sustainability procedures, and ensure effective system functioning?
Collective Action	The work that individuals and organizations have to do to enact the new practice. These processes are investments of *effort* made by participants.	Does the strategy facilitate the operational work necessary to support a new practice, which involves how people interact with each other and with the system, making sure everyone understands their roles and responsibilities, and how resources are managed to make the new system or process work effectively?
Reflexive Monitoring	The informal and formal appraisal of a new practice once it is in use, in order to assess its advantages and disadvantages and which develops users’ comprehension of the effects of a practice. These processes are investments in *appraisal* made by participants.	Does the strategy allow users to assess the new practice by looking at how it affects people and their work, and then deciding if any changes are needed based on what they find?

## Methods

We conducted a qualitative evidence synthesis using a rapid review approach. Rapid reviews use an accelerated process compared to standard reviews, allowing researchers to draw insights from scientific evidence in a timely and resource efficient way.^24^ This rapid review's protocol was registered on the PROSPERO database: CRD42021275000, and is reported following the enhancing transparency in reporting the synthesis of qualitative research (ENTREQ) statement.^25^ No amendments were made to the information provided at registration or in the protocol.

### Search strategy

The search strategy (see Appendix A) was adapted from the reviews conducted by Ross and Mair on the factors affecting the implementation of E-Health systems.^7,14^ For this review, the strategy was narrowed to search for studies on the implementation of EHRs only. Using the rapid review approach, we limited our search to only two electronic databases as more databases would increase the number of hits while diminishing the number of included papers. A systematic Boolean search method was employed on the 23^rd^ of June 2023 using key operators (such as OR and AND) truncated to find relevant literature. The search utilized two categories of keywords (see Appendix A), using Medical Subject Headings (MeSH) terms related to “Electronic health records” and terms related to “Implementation.” The search was initially conducted in PubMed and then adapted for CINAHL, using different indexing methods to suit each database and maintain the consistency of results. Both searches were limited to January 1st, 2010. The search strategy was refined through team discussions and the final search syntax is provided in Appendix A.

### Eligibility criteria

#### Inclusion criteria

Included studies were primary studies published between January 1, 2010 and June 23, 2023, of qualitative or mixed methods design, reporting on the implementation of EHRs in any health care setting, with end users of as study participants. Studies were included if they provided descriptions of engagement strategies used during implementation. Only studies in English were considered.

#### Exclusion criteria

Studies were excluded if their participants were only non-end users, or if they were reviews, meta-analyses, literature reviews or quantitative studies. By non-end users we mean individuals who do not directly use or interact with EHRs in their healthcare roles, which may include policy makers and managers. At final screening studies were excluded if they did not describe any engagement strategies used at any point during EHR implementation; there was no description of user involvement undertaken or attempted.

### Study selection

All identified studies (*n* = 1034) were checked for duplicates using the Zotero software. The remaining studies (*n* = 963) were double screened by title and abstract using the Covidence software by three members of the review team (CZ, SA and SM), guided by the inclusion criteria. Full text screening was conducted by the lead reviewer (CZ), screening the first 20 studies, while FG and JG independently screened the same batches to ensure consistency of screening decisions. Beyond this point, CZ conducted full text screening of the remaining studies and discussed the screening decisions with the senior co-authors (FG and JG) to ensure consistency and accuracy of this process. This led to the final sample of included papers (*n* = 41).

### Quality assessment

The standards for reporting qualitative research (SRQR) was used as a quality appraisal tool, the lead reviewer (CZ) assessed the quality of the articles with the input of senior co-authors (FG and JG). The SRQR consists of 21 items which provide SRQR.^26^ To adapt the SRQR for quality appraisal, we assigned a scoring system for each of the 21 items, scoring points for fully addressed items (1 point), partly addressed (0.5 point) and not addressed (0 point) (see Appendix B). For mixed methods studies, we focused exclusively on the qualitative parts of the findings and applied the SRQR accordingly. We used the total score as an indicator of the quality of the paper. Thus, from a possible maximum score of 21 (see Table 2), studies that had a score lower than 15 out of 21 were regarded as low quality. They were retained if they met the inclusion criteria but we were cautious in use of evidence from lower quality papers. As this is a novel use of the SRQR, we additionally assessed the quality of papers using CASP checklist (see Appendix C). The CASP ratings provided similar quality ratings but with a narrower range of scores, limiting differentiation between studies.

**Table 2.** Description of studies.

Author(s), Year	Country	Scale of implementation	Healthcare setting	Study design	Participants	SRQR score
Abramson, 2012	USA	Single unit	Academic affiliated, hospital-based ambulatory internal medicine practice.	Qualitative	Physicians	20
Alzghaibi, 2022	Saudi Arabia	National	Primary Health Care Centers	Mixed	General Manager, Head of Department, Deputy Head of Department, Software Developer, and Analyst	20
Bristol, 2018	USA	Single unit	Hospital	Qualitative	Nurses	20
Chang, 2016	Taiwan	Single unit	Hospital	Qualitative	Nurses	18.5
Chipps, 2020	USA	National	Multi-state hospital systems, academic medical centers, teaching, community hospitals in urban areas, specialty hospitals, and rural and critical access hospitals	Qualitative	Nurses with expertise in EBP, nurse informaticians (NIs), quality improvement specialists, advanced practice nurses and nurse administrator	18.5
Costa, 2022	Brazil	Multi-site	Prison Health Care	Qualitative	Doctors and Nurses	15
Craven, 2013	USA	Multi-site	Hospitals	Qualitative	CEOs, Directors of Nursing and IT, Vendors of systems, consultants working in the EHR market, Critical Access Hospital staff members, National policy makers, Researchers in clinical informatics and health information technology policy.	16.5
Creswell, 2011	United Kingdom	National	Specialist hospitals	Qualitative	Implementation team, Junior and senior nurses and doctors, allied health professionals and administrative staff	19
Cucciniello, 2015	Scotland	Single unit	Teaching hospital	Qualitative	Clinicians, nurses, Medical Director, Chief information officer (CIO)	18
De Vliegher, 2010	Belgium	Single unit	Home nursing organization	Qualitative	Head nurses, Administrators and Home nurses	18
Doyle, 2012	USA	Single unit	Family medicine training practice	Qualitative	Physicians	17.5
Fernald, 2013	USA	Multi-site	Primary care practice including family medicine, general internal medicine and general pediatrics	Qualitative	Family medicine, and general internal medicine practitioners, general pediatrics, REC staff leadership, REC staff, including executive staff, QI advisors, technical analyst.	17
Furlong, 2016	Canada	Single unit	Tertiary Hospital	Qualitative	Nurses	16.5
Gomes, 2019	Brazil	Multi-site	Hospital	Qualitative	Nurses	17.5
Grabenbauer, 2011	USA	Single unit	Hospital	Qualitative	Academic practitioners, private practitioners, hospital and academic administrators	20
Gui, 2020	USA	Multi-site	Hospital and Specialist centers	Qualitative	Physician Champions	20.5
Halas, 2015	Canada	Multi-site	Teaching clinics	Qualitative	Physicians, allied health faculty, and residents	17
Holden, 2011	USA	Multi-site	Hospitals and Outpatient clinics	Qualitative	Physicians, health faculty, and family medicine residents	17.5
McAlearney, 2013	USA	Multi-site	Hospitals	Qualitative	Physicians	19
McAlearney, 2015	USA	Multi-site	Hospitals	Qualitative	Managers, IT personnel and staff, physicians in practice and in training.	20
McCrorie, 2019	United Kingdom	Single unit	Academic Hospitals	Qualitative	Doctors and nurses of different grades, a hospital manager and a ward clerk.	20
Meigs, 2016	USA	Multi-site	Hospital	Qualitative	Physicians	17.5
Mishra, 2022	USA	Multi-site	Academic Medical Centers	Mixed	General physicians, family practitioners and pediatricians	18
Mohammed-Rajput, 2011	South Africa, Kenya, Rwanda, Lesotho, Tanzania, Uganda & Malawi	Multi-site	Resource constrained health care organizations (unspecified)	Mixed	Clinical providers, data managers, data assistants, system managers, administrators, nurses, supervisors, registration personnel, data entry personnel, reporting system developers, clinicians, physicians, researchers, vitals clerks, pharmacists.	16
Naratharaska, 2016	Thailand	Nationwide	Hospitals	Mixed	Physicians	17
Rantz, 2011	USA	Multi-site	Nursing home	Qualitative	Administrators, nurses and Nursing assistants	15
Rozenblum, 2011	Canada	National	Hospitals	Qualitative	Representatives from national and provincial agencies responsible for health information technology, quality/safety and public health agencies, health professional associations, and vendors of health information technology.	20
Scantlebury, 2017	United Kingdom	Single unit	Maternity unit	Qualitative	Midwives, Doctors and Health care assistants	21
Schnall, 2011	USA	Multi-site	HIV Case Management Agencies and Designated AIDS centers	Qualitative	Case Managers	16.5
Schoville, 2017	USA	Multi-site	Long term care facility	Qualitative	Director of nursing (DON), nurses (i.e. RNs and licensed practical nurses), and certified nurse aides (CNAs).	16
Ser, 2014	United Kingdom	National	Mental Health hospitals	Qualitative	Nurses, psychiatrists, social workers and allied health professionals	19
Sheikh, 2011	United Kingdom	National	Hospitals and Specialist centers	Qualitative	Junior and senior hospital managers, implementation team members and IT staff, junior and senior doctors and nurses, allied health professionals, administrative staff, patients and carers.	19.5
Sherer, 2015	USA	Single unit	Hospital	Qualitative	Physicians, clinical and non-clinical staff	17.5
Shiells, 2020	Belgium, Czech Republic and Spain	Multi-site	Nursing Homes	Qualitative	Occupational therapist, Social care supervisor, Nurse supervisor, Physiotherapist, Nurses, Care quality manager, Social worker, Home manager, Art therapist	19.5
Sieck, 2020	USA	Single unit	Academic medical center	Qualitative	Physicians	20
Sittig, 2020	USA	Multi-site	Clinics and Hospitals	Qualitative	Physicians, nurses, laboratory and radiology directors, information technology leadership, Chief Medical Informatics Officer (CMIO), and Chief Nursing Informatics Officer (CNIO)) and supporting staff, administrative leaders, and administrative support staff.	15.5
Swanik, 2019	USA	Multi-site	Dental specialties (private practice, hospital practice, teaching, and public health practice)	Qualitative	Dental faculty staff	16.5
Terry, 2018	Canada	Multi-site	Family health practice	Qualitative	Family physicians, nurses, pharmacists and social workers and included practitioners (Advanced EMR users)	20
Topaz, 2016	45 countries	Multi-site	Dental hospital	Mixed	Nurses, health informaticians, pharmacists and biomedical engineers	16
Upadhyay, 2022	USA	Multi-site	Trauma hospitals, Academic medical centers, Medical clinics, Home health centers, and Small hospitals.	Qualitative	Physicians, hospitalists, nurse practitioners, nurses, and patient safety officers	17.5
Watson, 2023	USA	Single unit	Hospital	Qualitative	Clinical informaticists, human factors engineers, business process analysts, process architects, requirements analysts, and business process re-engineers.	16

### Data extraction

The lead author (CZ) extracted data from eligible studies (*n* = 41) into an Excel spreadsheet and word document. The details from each study were recorded and these include the names of authors, year of publication, participants, design, study setting/context, intervention characteristics, description of engagement strategies used and user perceptions on engagement strategies (see Tables 2 and 4). After this stage, JG and FG met with CZ to review the consistency of the extraction. All included studies featured engagement strategies, but only those providing detailed insights were incorporated into Table 4. These offer rich information on strategy implementation and user perceptions. Studies with less depth are only included in the results section text (See next section for how this was managed during analysis).

### Data analysis

The results of all studies were inductively coded using descriptive themes in NVivo. Themes were grouped by type of engagement strategy (see Table 3). The coded data from richer studies was interrogated to create Table 4 which reports on the context of implementation (setting and scale of implementation), engagement strategies that were used at each stage of implementation (before, during and after implementation) and the success or failure of the strategies as reported in the paper. Papers reported success/failure as perceived by their research participants. We used the NPT constructs (coherence, cognitive participation, collective action and reflexive monitoring) to identify the reasons for the success or failure of each engagement strategy.

**Table 3.** Description of engagement strategies.

Engagement strategy	Description	Studies
Communication of policy and vision	This involves articulating, through various means, a clear and compelling vision or plan for the implementation of EHRs, which may occur along with outlining policies that guide their implementation, ensuring alignment, understanding, and support from all stakeholders in the healthcare environment.	^28–37^
Preparation for implementation	This is characterized by an early involvement of end users in system selection and co-design, along with the establishment of an implementation team, which aims to foster user engagement, ensure system alignment with operational needs, and facilitate a coordinated approach for the successful integration of systems within the organization.	^28–30,37–46^
Training and education	Involves the use of formal training methods to equip end users with the necessary skills and knowledge required for effective use of EHRs. This could include provision of learning material, on-site formal sessions, simulated practice and tailored training for specific user roles.	^29–32,34,35,38,41,42,44,46–63^
On-demand technical support	This refers to the availability of IT personnel, online self-service systems, and informal learning opportunities through consultations from colleagues who are proficient users, to provide timely and varied assistance for users encountering technical challenges in EHRs usage.	^29,30,34,40,42,47,51,53,54,58,64–67^
Ongoing engagement for user informed feedback	This entails a continuous process of communication with end users, encouraging feedback, and facilitating suggestions for modifications to the electronic health records (EHR) system, ensuring a dynamic and responsive approach to meet evolving needs and preferences.	^28,30,50,57,64,68^

**Table 4.** Evidence synthesis of results from 16 studies.

Study	Scale	Stage	Engagement strategies	Perceived successes, challenges and solutions to strategies	NPT constructs				Perceived outcomes
					Coherence	Cognitive Participation	Collective Action	Reflexive Monitoring	
Cucciniello, 2015	Single unit	Before	**Preparation for implementation** Users were involved in choosing the system through a workshop where they chose from different software vendors	Successes Due to the user-centered approach, users became advocates of the system and were actively involved throughout the implementation process	Yes	Yes	Yes	Yes	Successful
		During	**Training and Education** An implementation team trained super users. Key users and super users were appointed as local facilitators to support and train other users.	Successes Super users’ were useful as users were no longer required to leave their wards for training or to get help.	Yes	Yes	Yes	Yes	Successful
			**On demand technical support** Clinical advisors internally recruited to assist with supporting implementation and were responsible for conceptualizing, developing and administering training and service delivery, and providing technical input. A team was established that met once a month to provide help where needed.	Successes The support started informal but grew past implementation to picking up on user mistakes and showing them how to correct them.	Yes	Yes	Yes	Yes	Successful
			**Ongoing engagement for changes required** Implementation was structured, starting with the most relevant functions in the facility, followed by piloting additional functions in single wards in order to test them and to get feedback from staff working on the selected wards.	Successes This helped make any adjustments based on results and the progress made when using the system.	Yes	Yes	Yes	Yes	Successful
Gui, 2020	Multi-site	Before	**Training and Education** User training prior to going live	Challenges Only offered to physician champions Training was too generic, and not tailored to depts’ contexts Took place after the champions had to customize the system No live or simulated practice Solutions Champions developed training with simulated environment for own department	Yes	Yes	Yes	Yes	Successful
		After	**On-demand technical support** “At the elbow” support after going live	Challenges Support was insufficient Champions tried to communicate with system builders but failed Solutions Champions sought help from super users or hired in external people Champions facilitated peer support	Yes	Yes	Yes	Yes	Successful
Chipps, 2020	Multi-site	During	**Preparation for implementation** Nurse Informaticians (NIs) acted as advocates for system use. Some NIs were embedded with the IT team while others were in the nursing department.	Successes NIs were highly valued. Challenges A high volume of work shifted to the NIs and they were understaffed.	Yes	Yes	Yes	No	Successful
		After	**On-demand technical support** An online self-service request system for staff users to make contact developers on EHR issues. This system was posted on the organization's internet page so all staff had easy access to it	Successes Self-service request system was viewed favorably Challenges High volume of tickets due to the organization's size led to slow response. Some users submitted a request to an immediate supervisor with limited success	Yes	Yes	No	Yes	Successful
Holden, 2011	Multi-site	During and After	**Training and Education** There was formal training for users followed by informal support where users learnt from their colleagues	Successes Initial formal training was depicted favorably by some, Many physicians said they were better able to use EHR from talking to and observing colleagues using EHR. Challenges Training was often insufficient and the classroom style was ill suited for users Physicians preferred hands-on, in-person training provided in practice rather than formal training	Yes	Yes	Yes	No	Successful
			**On demand technical support** Technical support facilitated use both in the initial days and weeks of EHR and afterwards.	Successes Support staff were generally perceived as knowledgeable and helpful Physicians were most appreciative of one-on-one, on-demand support during actual care scenarios. Challenges Support staff were unavailable sometimes (off hours and holidays).	Yes	Yes	Yes	Yes	Successful
De Vliegher, 2010	Single unit	During	**Training and Education** Home nurses received manuals prior to actual use of ENR. There was no manual in one of the departments. All users received 2 sessions of 4 h education. First session was theoretical. In second session nurses received a ﬁctitious list of patients to try out the different screens.	Successes Manual was appreciated as users could always fall back on it when they needed help. Manual was necessary, especially on weekends, when it is hard to contact someone. Education was viewed as necessary and useful by users	Yes	Yes	Yes	Yes	Successful
			**On demand technical support** Nurses ﬁrst contacted the administrator with queries. If they could not resolve the query it was passed to the head nurse, and then if necessary to the information technology (IT) manager.	Successes Users reported having adequate support and problems were solved usually on the same day	Yes	Yes	Yes	Yes	Successful
Sieck, 2020	Single unit	During	**Training and Education** Tailored training was provided to cater for different user roles and physician representatives shared training strategies based on what was done in other units. Users also learnt from colleagues through observations and sharing of tools.	Successes Tailored training helped users to adapt as it was more specific to their roles Learning from colleagues improved the initial training, which was often generic.	Yes	Yes	Yes	Yes	Successful
		After	**Ongoing engagement for changes required** Department representatives participated in EHR-committees. System changes were communicated to users primarily via emails	Successes Physicians were represented and included in the modifications and decision making process	Yes	Yes	Yes	Yes	Successful
				Challenges Some users preferred periodic updates for massive updates communicated via staff meetings Emails were too frequent causing fatigue and users ignored some of the emails	No	No	No	No	Unsuccessful
Schoville, 2017	Multi-site	Before	**Preparation for implementation** Leaders in one site involved users in vendor selection.	Successes Leadership was available for support in all sites from the beginning, and there was good teamwork.	Yes	Yes	Yes	N/A	Successful
		During	**On demand technical support** Super-users were appointed at all sites. These were individuals who were most knowledgeable about the system and available to assist with troubleshooting users’ calls and questions	Successes Super users were useful and assisted with troubleshooting users’ calls throughout implementation.	Yes	Yes	Yes	Yes	Successful
			**Training and Education** Education and training sessions in formal classroom settings, competency testing, and a practice playground environment. Information was also shared through education manuals, direct communication, e-mails, and cheat sheets.	Successes Communication strategies were mostly viewed as positive and supportive by the users. Challenges Training was insufficient and inconsistent as educator was not a nurse and did not understand user workflow Users needed real scenarios with training. Staff at one site reported threats of punishment being used with problem solving.	No	No	Yes	No	Unsuccessful
		After	**Ongoing engagement for changes required** Auditing across sites to keep leadership informed about EHR use. Some sites’ strategies included bi-directional feedback, seeking it in staff meetings, e-mail, and open-door policy with employees.	Successes Feedback enhanced problem solving especially when facility leadership communicated the outcome of the comments.	Yes	Yes	Yes	Yes	Successful
McAlearney, 2013	Multi-site	Before	**Preparation for implementation** EHR adoption decision was communicated across all sites and the vendor selection process was done by management and IT personnel	Challenges Lack of early physician buy-in, with several sites noting that, ideally, the physicians themselves should drive the EHR adoption decision as opposed to the health system. Users should have been part of the vendor selection process to ensure that the product selected could support their clinical needs/preferences.	No	No	No	No	Unsuccessful
		During	**Training and Education** Formal training was provided prior to going live. All physicians were required to participate. In one organization, physicians had mandatory e-learning requirements and then had to demonstrate competency with basic EHR functions before they could use the new EHR system for patient interactions.	Successes The most successful trainings were conducted within a few weeks of go-live. Physicians found it useful to get an opportunity to “play around” with the system prior to going live Although time-consuming, many sites reported that having physicians abstract their own charts and “preload” data was an important part of the training process.	Yes	Yes	Yes	Yes	Successful
			**On demand technical support** Support was provided after the going live period and most sites offered on-site IT support (vendor and/or internal IT) during the go-live period to answer questions and help troubleshoot on the spot.	Successes The support team was capable and an essential facilitator In-person support was particularly well received by physicians anxious about the transition to the EHR.	Yes	Yes	Yes	Yes	Successful
Sittig, 2013	Multi-site	Before	**Preparation for implementation** User testing was done prior to implementation, a process that was led by vendors	Successes Users shared that they were able to test the system thoroughly Challenges Only a select few did the testing User feedback was largely ignored, thus the implemented version came with malfunctions	No	No	No	No	Unsuccessful
		During	**On demand technical support** Personnel to support EHR implementation and use. This was in the form of a multidisciplinary team of people capable of assembling all the required hardware and software components of the EHR, configuring these components, and deploying them throughout the organization. They worked with health facilities a year before going live	Successes Users regarded the team as key personnel to safe EHR use	Yes	Yes	Yes	Unknown	Successful
Terry, 2018	Multi-site	During	**On demand technical support** Advanced electronic medical record (EMR) users as an agent of change. They acted as motivators of change, mobilizing team members to come on board with activities such as data standardization and workflow changes, which would support advanced EMR use.	None mentioned	Yes	Yes	Yes	No	Successful
			**Training and Education** Electronic medical record training and education.	Challenges Training and conferences were only offered to advanced users Solutions Participants recommended non-advanced EMR users receive training and attend user conferences, and to consult with peers who were more advanced. Some suggested that newer users attempt problem solving within the EMR, thus engaging in experiential learning.	No	No	No	No	Unsuccessful
Scantlebury, 2017	Single unit	Before	**Communication of Policy and Vision** Policy changes leading to new audit requirements	Challenges Vast amounts of “top-down” in policy change Long-term staff, impacted by a previous failed system, were skeptical and did not differentiate between the two systems.	No	No	No	No	Unsuccessful
		During	**Training and Education** Management offered supplementary resources, extra training, “lessons learned” emails, and electronic guidance for complex tasks	Challenges The delivery of training was too simplistic and dogmatic Training was inconsistent and varied in content and duration (ranging from none, a single 30 min or all day sessions) Timing of training was too far in advance of, or after implementation)	No	No	No	Yes	Unsuccessful
			**On demand technical support** A support team of super users was appointed to help staff in using the EHR	Successes Super users were useful particularly for those with poor computer literacy. Challenges The team had limited availability (Weekday office hours only)	Yes	Yes	Yes	Yes	Successful
Creswell, 2011	National	Before	**Communication of Policy and Vision** The government shared a vision of national EHRs via targeted communication from the national bodies to hospital managers, and to users, stating expectations of the staff and the beneﬁts of the system. This was done to mitigate a recognized risk of users refusing to use the new system and to win over skeptical staff	Successes Users generally bought into the vision of EHRs. Challenges Some hospitals did this effectively and others didn’t, leading to an enforced use of the system by withdrawing the paper system	No	No	Yes	No	Unsuccessful
			**Preparation for implementation** National bodies withdrew existing paper systems to force use of EHR.	Challenges Hospital management trying to “sell” software that it had not chosen to clinical and administrative staff. Early release software had limited functionality making it difficult to demonstrate the product to potential users	No	No	No	No	Unsuccessful
		During	**On demand technical support** Centrally appointed national, and locally appointed clinical, champions	Successes Locally appointed champions were effective communicators between management and clinic groups and were valued by system users and hospital management.	Yes	Yes	Yes	Yes	Successful
				Challenges The centrally appointed national champions lacked credibility and couldn’t facilitate effective two-way communication between mx and users.	No	No	No	No	Unsuccessful
Rozenblum, 2011	National	Before	**Communication of Policy and Vision** National bodies shared an E-health plan, a comprehensive national approach for health information technology	Successes Some parts of the E-health plan was generally viewed as successful Challenges There was a lack of an e-health policy to foster effective strategies for adoption by clinicians. The plan had inadequate attention to clinicians as key users of EHRs, which was viewed as critical It needed to be more flexible and adaptive to accommodate user feedback	No	No	No	No	Unsuccessful
Ser, 2014	National	Before	**Communication of Policy and Vision** Local management communicated the vision of national EHRs to users	Challenges Hospital leaders: Did not understand the views of the mental health clinicians, who are the end-users Hadn’t communicate successfully to their staff how EHR systems fit into a wider vision of improving patient care and efficiency in the NHS	No	No	No	No	Unsuccessful
		During	**Training and Education** User training prior to implementation	Challenges Lag time between training and implementation was too long Training not tailored sufficiently to users’ needs	No	No	No	No	Unsuccessful
McCrorie, 2019	Single unit	Before	**Preparation for implementation** User preparation involved road shows for awareness, on-site demos, training, e-learning, and additional training for designated “EHR friends” or “super-users” for the “go-live” weekend.	Successes The preparation allowed users to identify areas of concern prior to implementation Challenges Some participants (those not involved in EHR set-up) were concerned that the potential for intelligent problem solving was missing. No direct communication with developers. Perceived complex chain of command hindered customization transparency in the system.	No	No	Yes	No	Unsuccessful
		During	**Training and Education** Training was done as part of the preparatory efforts for implementation	Challenges Training was not enough, or was found to be too intense or generic. Training inadequately addressed generational differences in computer literacy. Limited access to play domains.	No	No	Yes	No	Unsuccessful
Bristol, 2018	Single unit	During	**Training and Education** The organization provided users with hardware and training	Challenges Lack of training before implementation, thus it came late Shortage of hardware (computers) to facilitate training There was poor organizational support of the training process	No	No	No	No	Unsuccessful
		After	**Ongoing engagement for changes required** There was communication between EHR users and EHR designers	Challenges The organization overlooked the unique perspectives of nurses. Nurses were frustrated due to a lack of attention from EHR system designers, leading to systems poorly equipped to address workﬂow issues.	No	No	No	No	Unsuccessful

Through a qualitative comparative analysis (QCA) process, we compared the studies to identify what combination of context and engagement strategies resulted in successful engagement (see Table 4). The QCA is a method for identifying the configurations of conditions that lead to specific outcomes.^27^ A narrative synthesis was then written, drawing in data from the papers beyond Table 4 to provide diverse examples and avoid overlooking disconfirming results.

## Findings

### Description and overview of studies

**Figure 1.**
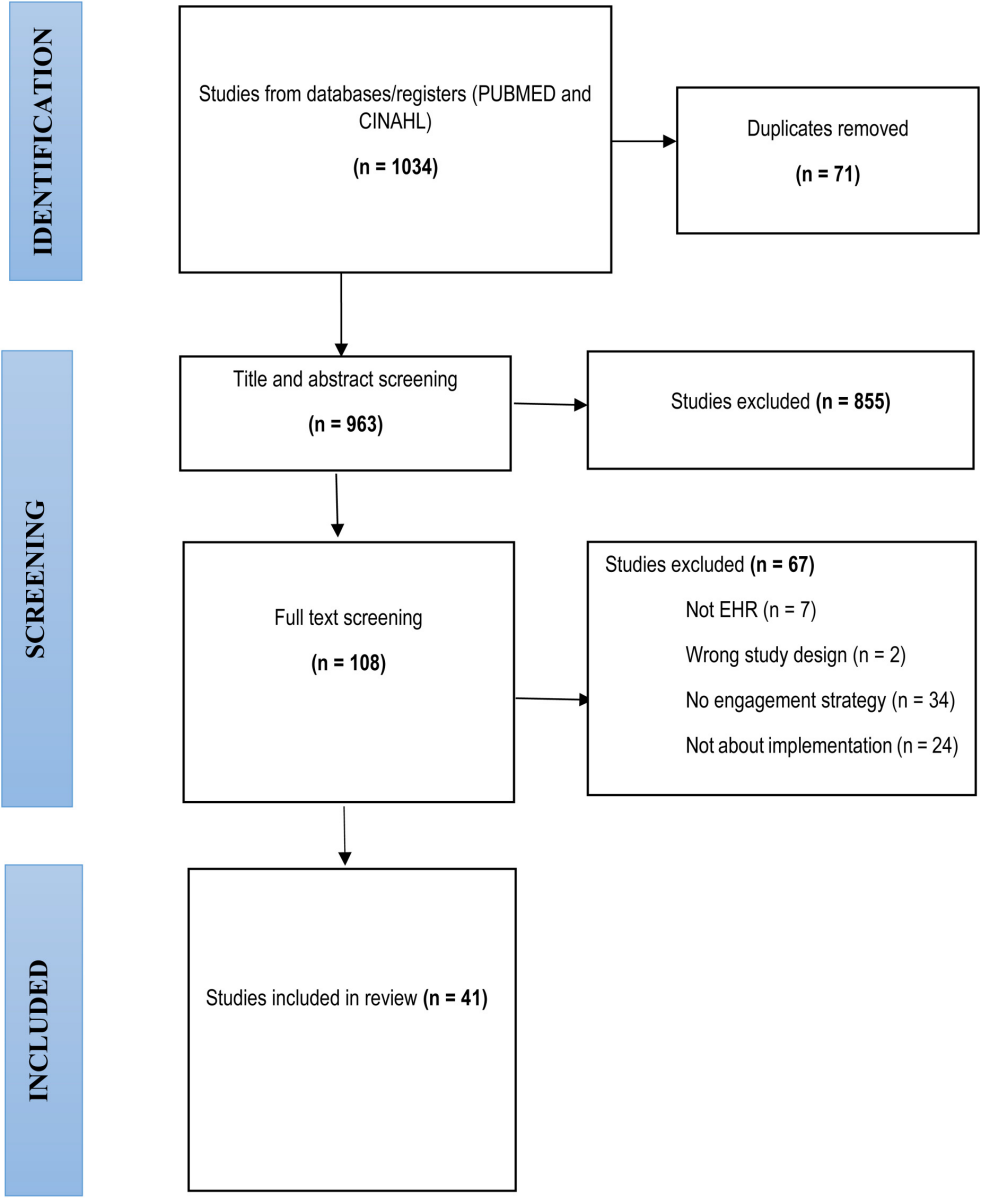
PRISMA flow diagram.

The search yielded 1034 results, reducing to 963 after removing duplicates. From these, 108 were remaining after title and abstract screening, reducing to 41 after full text screening (see Figure 1), with 36 qualitative and 5 are mixed methods studies. Quality assessment resulted in 21 high-quality (18–21/21) studies, 17 average-quality (16–17.5/21) studies, and 3 below-average studies (<16/21). We included all studies but found higher quality papers had richer and more useful data for our review.

Of the 41 studies, 38 were from high-income countries, 2 from middle income and 1 from a low-income country (see Table 2). Additionally, 13 studies were conducted in single units/facilities, 18 were multi-site or regional implementations, 7 were national implementations, and 3 were multi-national implementations. 23 studies were conducted in hospital settings, while the remaining 18 were spread across mental health, maternity, long-term care, nursing homes, ambulatory, teaching clinics and HIV care facilities. Among the 41 studies, 22 primarily involved nurses as participant users, while the other 19 had participants from various professions, including physicians, doctors, dentists, pediatricians, pharmacists, administrators, data capturers, and mental health practitioners. Of the 41 studies, 22 involved non-user participants: managers, software developers, engineers, heads of departments, case managers, quality improvement specialists, policy makers, researchers, software vendors, business and process analysts, safety officers, information officers and process architects.

Table 3 gives a list of the all the identified engagement strategies, descriptions of each strategy, and references to studies reporting these strategies.

### Before implementation

Engagement strategies used at this stage aimed to prepare facilities and end users for implementation beforehand.

#### Communication of policy and vision

Policies at national level were perceived as an effective way of communicating intention across multiple facilities and to foster buy in and collective action for stakeholders at various levels as one study reports: “*You need upper-management buy-in and buy-in from all department heads, including HIM director or materials management, not just top administration. Clinical and operational leadership must be engaged fully in the process*.”^28^ In most cases of national and multi-site implementation, policies and visions were generated at national level but facility leaders proactively made policy changes and provided support to users.^29–31^ Several studies show facility leaders playing this role in preparing and engaging users at this stage.^30–34^ Local facility managers should be able to engage end users according to their own managerial insights and organizational needs to ensure that users can make sense of the new system. Communicating the vision requires leaders to manage expectations for users as one study highlights: “*This is an organizational change project not an IT implementation project, and it will rise and fall on how much the leaders—all the leaders not just the CEO—understand and set and guide expectations*.”^28^

However, in some cases policy and vision were communicated from the top but lacked engagement with local leaders leading to poor communication with end-users.^32,34–39^ For instance, where implementation was led by national structures without engaging users and leaders from local organizations, the vision was perceived as poorly communicated.^34^ In such cases, communication was in the form of a directive, which limited facility managers’ autonomy to tailor implementation for their organizations. One study shows how this led to poor coherence and cognitive participation, as users perceived the EHRs as imposed: “*It's getting the users to understand and the hospital to understand that the system is theirs and they own it…The danger is they feel it's thrust on them and they have to use it*.”^32^ Thus, local management struggled to communicate the policy and vision when national bodies used a top-down engagement approach. In another study, national bodies later tried to address this issue after users showed resistance: “*I think it's about what are we communicating, how we communicate that, what are we saying our expectations are to staff about what are the beneﬁts of this system to patients and to staff*.”^34^ This was done through communication strategies, emphasizing anticipated benefits, training, appointing clinical leads, and seeking user input in system design.

#### Preparation for implementation

Engaging end users prior to implementation was deemed important, with local leaders playing a crucial role in translating the implementation vision into practical tasks.^30,40^ This enables sense-making, helping users understand the coming changes and their roles in it. One study reports how multiple sites minimized workflow disruption by conducting a “readiness to change” assessment at facility and individual levels.^29^ This involved assessing issues such as current user IT usage in facilities and computer literacy to inform implementation plans. One study reports how preparation for implementation should involve collective action: “*Assembling the right team—usually the practice manager, a clinician, and staff members—to plan and implement this work was essential. We have put a team together that involves every area of the practice*.”^41^

As shown in Table 4, early preparation involves leaders proactively engaging users early, and involving them in vendor selection, planning and decision making stages.^29,30,34,42–44^ This secures cognitive participation, fostering collective work towards a goal as one study reports: “*Across sites, informants emphasized the importance of early physician buy-in, with several sites noting that, ideally, the physicians themselves should drive the EHR adoption decision as opposed to the health system. Early and strong physician buy-in was deemed critical in order to ensure successful implementation and minimize resistance*.”^29^ Another study showed how early user involvement generates buy-in: “*participatory process in selecting the EMR system represents a distinctive way to generate and improve commitment to the project within the organization. Conversely, if this process is imposed, it can generate user frustration and have a negative impact on the implementation process and on the overall use of the system*.”^43^ Several studies showed that the overall benefit of early user participation is that users can select a system that meets their needs.^29,30,38,45^ One study suggests that learning from facilities with similar systems can further inform user choices: “*Take a group of staff to visit another hospital that has the system you’re considering; observe how the system works, and ask to hear their stories about system build and use*.”^28^

In cases of poor preparation, poor user cognitive participation was also noted. For instance, when national bodies imposed EHRs on users, local management struggled to prepare their facilities because they perceived it as an attempt to promote software that was not chosen by intended users.^34^Such engagement allows early feedback from users, through reflexive monitoring. In one study, users tested the system but their feedback was largely ignored leading to a rollout of a system that malfunctioned.^45^ Similarly, in one facility, extensive user preparation included awareness road shows, on-site demos, training and e-learning before going live, but the system malfunctioned poorly as users could not communicate with developers for intelligent problem-solving.^46^

### During implementation

Engagement strategies used at this stage were aimed at supporting end users and managing challenges during the implementation process.

#### Training and education

Training and education were frequently reported in the findings. Studies shared user insights on training methods, timing, frequency, content and the delivery of training.^37,47–49^ Training that was reportedly successful was delivered a few weeks before going live,^29^ with one study cautioning against delaying EHR rollout after training as it may lead to users forgetting what they learnt.^32^ Training that came too early or too late after the rollout was reportedly unsuccessful.^36,50^ While timing was important for initial training, users felt that refresher training was particularly helpful following system upgrades.^42,51,52^

Studies report various training methods: formal course training, simulations and practice, providing user manuals and leveraging highly trained users to train colleagues.^40,46,53,54^ Hands on and simulation training were favored by users because they allowed users direct interaction with systems.^29,54,55^ Users describe its usefulness in real-work situations: “*[The training should be] more applied by focusing on a patient scenario, to facilitate learning how to use the system safely and efficiently during a patient visit*.”^56^ In cases where users had varying levels of computer literacy, training started with basic computer education.^54,57^ In a nursing home study, users found it beneficial to supplement theoretical training sessions with manuals as they provided a convenient and quick reference: “*it is reassuring to know that you can always fall back on it [manual] when you have doubts about putting things in the right screen*,” while another stated: “*a manual is necessary, especially in the weekends, when it is hard to contact someone*.”^58^ In other cases, training was supplemented by emails on lessons learnt and guidance for complex tasks.^36,59^

Some users, did not favor formal training, and found it too generic to just sit and receive information on how to use the system from a trainer.^54,60,61^ In Table 4, formal training was successful when supplemented with peer support and learning.^43,47,48,57^ Users also preferred hands on training and learning from peers more: “*[Training with technical support] was pretty useful [but] experimentation is number one and then being taught by colleagues*.”^62^ In such cases, proficient users who were peers and colleagues facilitated training tailored for their context for non-proficient users.^29^ This eliminated the need for users to leave their wards for more training or support.^43^ In one study, physician champions through reflexive monitoring noted that the training was too generic and developed a tailored simulated training environment for users before going live, which was well received.^47^ However, in two studies, training was only offered to advanced users, neglecting the broader user base and leading to poor training outcomes among users.^46,63^

Users preferred tailored training because it catered to their unique roles and workflows.^33^ Conversely, where training was not tailored, users found it irrelevant: ‘‘*If you were an admin person, you’ve got a certain type of training…And so, you had people sitting in training session where maybe 50% of what was being trained was not really applicable to their job*.”^32^ Thus, failed training efforts were typically attributed to generic and untailored training approaches,^32,63^ which discouraged cognitive participation. Other challenges in training included inconsistent and insufficient training,^44^ as one study reports: “*Participants found EHR training to be superficial and of insufficient duration. Some obtained training for a few hours before using EHR for clinical activities. Some were trained as they performed clinical activities, and others got trained over the phone*.”^49^ Users noted that training was poor when delivered by individuals unfamiliar with the workflow or the specific work of users as they perceived that such trainers could not effectively bring coherence and cognitive participation among users.^30^ Users felt that training was also unsuccessful when imposed on them. In some facilities, users were reluctant to take time to learn until necessary, as some managers mandated training, requiring users to demonstrate competency before EHR use.^29,30,46^ However, this approach was perceived to be too intrusive and less effective in aiding users to make sense of EHRs.

#### On-demand technical support

Users appreciated real time, hands on, in person and on demand support during actual care situations.^30^ Some multi-site studies highlighted the effectiveness of real-time support,^51,54,64^ with one study emphasized the value of on-site developer assistance due to the convenience of developers’ prompt availability.^51^ In this case, the on-site developer was available for user support throughout their work shifts.^51^ Another study achieved this by establishing direct contact with off-site developers who could remotely assist users: “*staff could write notes and feedback any problems directly to the developer, who also had remote access and could look into any issues quickly*.”^54^ However, slow responses could led to frustration, especially during urgent situations as reported in one large organization: “*Essentially, anyone can submit a ticket but as far as the response, I guess there are thousands of tickets so the ability of the organization to respond personally, you always get an automated response*.”^64^ As a workaround, some users submitted their requests to their immediate supervisors, who escalated it up the “*chain of command*.” One nursing home required nurses to sequentially contact the administrator, head nurse, and IT manager to address problems^58^ and issues were resolved on the same day.

On-demand support was also provided by super users, physician champions, and nurse informaticians.^36,39,64,65^ Physician champions were perceived to be effective in providing localized support during critical periods, a month or two after the system's implementation. Similarly, another study made use of nurse coordinators for this task: “*Our nurse coordinators who served as super users…really knew the system and were adept at helping their colleagues and physicians*.”^47^ In this study, external people were also hired to provide extra support on a part time basis.^47^ Where nurse informaticians (NIs) were used, they initially facilitated implementation and continued to provide user support after, with some NIs directly embedded with the IT team, and others in the nursing department.^64^ Although NIs were understaffed, users found them useful in promoting system use. In other cases, similar roles were fulfilled without specific titles, where a team of proficient clinicians acted as knowledge resources and were strategically positioned in wards.^34,39,43^

On-demand technical support was perceived as more effective when provided by peers as they leveraged existing relationships and were often influential enough to garner the trust of other users. However, when these roles were imposed or incompatible with user workflow, there was limited success.^34^ Several studies underscore the importance of providing well trained support personnel.^29,53,66^ In one case, IT support personnel were slow in responding to problems causing delays and user frustration: “*IT personnel need to quickly assist us in resolving problems… If the problem remains the same, how can you expect us not to have negative emotions toward this system?*.”^67^ Poor support led to users disengaging: “*In fact, we are not uncooperative with the hospital's policy to use technology. We only hope for more support during the adaptation period. Our workloads remain the same, and adapting to the new system is an additional task, which is exhausting*.”^67^

### After implementation

Strategies implemented at this stage were aimed at sustaining user involvement by addressing ongoing needs and improving the system.

#### Ongoing user engagement

Ongoing engagement ensures continued use and improvement of EHRs through reflexive monitoring, especially when introducing system upgrades, changes and adding new features as users need to be informed about these changes.

Users perceived that channels for continuous user feedback are useful for making informed improvements, with effective channels involving bi-directional feedback.^28^ In one study, sites used different engagement approaches for bi-directional feedback, with some through staff meetings, emails, an open-door policy, and by allowing direct communication with the Director of Nursing (DON).^30^ Users from this study found this to be an effective way of engaging as leaders would relay back the outcomes of their input back to them. Another study showed that effective ongoing engagement was through regular meetings of physicians from various specialties in EHR-focused committees.^57^ This approach, through the collective action of various users, ensured the input of diverse perspectives for planned upgrades and changes.

Facility leaders played an important role in establishing communication channels, and users reported favorably towards positive communication: “*Leadership communication strategies about implementation were mostly positive and supportive to the user…All groups reported a range of communication strategies, including education manuals, direct communication, e-mails, cheat sheets, and auditing*.”^30^ In another study, nurses who were intermediaries during implementation also facilitated this communication: “*We have frequent updates [EHR system], guided by nurses who have become part of the program. Our system therefore has become close to exactly what we need…I contact one of those nurses who usually make [sic] it happen*.”^50^

Although updates were appreciated, users preferred receiving periodic updates, communicated through regular staff meetings with developers: “*What I think would be really helpful is sort of periodic updates in various forms. If there's going to be a massive change or update…if they could come to our division meeting, department meetings, probably division meeting, or even our clinics*.”^57^ Some users preferred less frequent email updates and admitted to ignoring non-urgent emails due to email fatigue: “*I hate to say it that way, because we get, there's email fatigue, too…A lot of times I don’t even open’em. I delete a lot of stuff. You just gotta’ focus on stuff that seems relevant for the moment to get you through the day*.”^57^

However, users in large organizations faced challenges with complex organizational layers to provide feedback: “*Now that we are part of a larger system, making those changes, just go into layers and layers of people to get those changes*.”^64^ Thus, ongoing engagement can be complicated by organizational size and can lead to user frustration.^68^

### Use of engagement strategies

Table 4 shows that successful implementation requires the use of multiple strategies concurrently. A contrast can be made between the Cucciniello^43^ study, which used 4 strategies and can be seen as more successful, against the Bristol^50^ study, which used 2 strategies with less success. In the Cucciniello study,^43^ implementation was more successful because users were involved and prepared early, offered training and support through peers, and a phased implementation approach was used to allow for real time adjustments during rollout. This ensured user coherence and cognitive participation through early involvement, followed by collective action and reflexive monitoring through peer led training and support. In contrast, the Bristol study^50^ reports only training and ongoing engagement after rollout, leading to a poor implementation due to poor coherence and late training, rendering it ineffective.

Users perceived that another important success factor for engagement strategies is the capacity for reflexive monitoring. Studies that were reportedly successful were able to revise, and adapt where necessary due to feedback from user groups such as super users and champions,^43,47^ representative groups,^57^ online platforms^64^ and direct engagement with users.^30^ The constant monitoring and dynamic responsiveness led to a more successful use of engagement strategies and a more resilient implementation.

## Discussion

Our findings show that effective communication of policy and vision is crucial for user buy-in as a top-down approach without user participation may result in poor outcomes. Preparation for implementation and early user involvement in decision-making processes are effective engagement strategies prior to implementation. This involves assembling a diverse and representative team to facilitate implementation. Tailored training, especially through simulated practices, accommodates diverse user roles, workflows, and user literacy. However, critical factors for training effectiveness include timing, content and methods used. During rollout, on-demand support from technical personnel and proficient peers is crucial engagement to promoting EHR adoption. After implementation, ongoing engagement sustains a successful EHR adoption and optimization process through user feedback and active participation.

Similar to our findings, reviews emphasize the importance of user involvement for user readiness and better adoption.^69^ Reviews report on the effectiveness of individual or clusters of engagement strategy reported in our study. Policies are crucial in fostering user readiness. One review shows that in developed and developing countries, policies were key determinants of readiness for EHR implementation.^70^ Involving users early in the co-design of EHR systems has proven effective in improving implementation by ensuring the system caters for user needs, minimizing workflow disruption.^71^ Another review demonstrated how tailored training, before, during and after EHR implementation is effective, especially when it includes super users, peer to peer and a mix of different training methods.^72^ Successful adoption of digital technologies in clinical settings often hinges on organizational support, including that of personnel such as physician champions.^73^ Ongoing formal programs of peer support groups, and online tools was reported to be an effective way of continuing to engage users.^74^

WHO resolutions on digital health implementation encourage governments to develop strategies that guide implementation.^6^ While global EHR adoption is rising,^75^ many studies in this review focus on high-income countries (see Table 2). Several middle-income countries such as China, Brazil, India, and Mexico have initiated their EHR adoption, but most of them are still in early stages primarily due to financial and technological challenges.^5,76^ This review draws insights from counties that have implemented EHRs to aid those in early stages. Low- and middle-income countries face challenges when implementing an EHR challenges including financial constraints.^77^ This review synthesizes learning that can contribute to implementation success and the avoidance of costly failures of implementation such as that in England in the 2000s.^78^

Successful implementation is important because of the stress caused to health-workers during implementation due to heightened uncertainty.^79^ Effective engagement strategies can reduce this stress.^80^ Evidence suggests that user stress can be reduced through tailoring EHRs to clinicians’ needs, conducting usability testing and providing ongoing support.^81^ High levels of EHR related stress have been linked to an increased intent among clinicians to leave their clinical practice.^82^ Low- and middle-income countries already face challenges in retaining their clinical workforce.^83^ User engagement during implementation of an EHR can help retain the clinical workforce. Engagement strategies remain necessary even after implementation to keep clinicians motivated and promote mature use of EHRs.^84^

### Implications for policy and practice

This study underscores the importance of user engagement in implementing EHRs and identifies the effective strategies along with the underlying reasons for their success. Inclusion of user engagement within policy can enable implementers to give priority to user engagement during implementation. This evidence review can inform implementers’ decisions on how to engage users, when and why. This is particularly important for low- and middle-income countries now considering the roll out of EHRs to support universal health coverage, where costly failure needs to be avoided.

### Recommendations

The table below outlines recommendations drawn from the study findings on effective engagement strategies for EHR implementation, offering guidance on when and how to apply engagement strategies (Table 5).

**Table 5.** Recommendations

Before implementation	Communication of policy and vision
	Effectively communicate the vision of EHR implementation through a two-way engagement with users and proactively implement policy changes beforehand.
	Preparation for implementation
	Involve users in decision-making stages and create designated teams from user ranks to aid in the implementation process for increased buy-in. Facility leaders should guide the implementation process and translate the vision into practical tasks to users.
During implementation	Training and education
	Training and education are necessary and should be timely, incorporating hands-on and simulation methods, and tailored to users’ roles Successful training involves avoiding generic and untailored approaches, considering diverse user needs, and supplementing formal training with peer support.
	On-demand technical support:
	Implement post-training support mechanisms, including real-time assistance, to enhance user experience and adaptation. Establish specific roles such as super users, physician champions, and nurse informaticians to provide on-demand support, prioritizing peers for effective assistance. These roles should be filled by individuals who are well trained and prepared.
After implementation	Ongoing user engagement
	Establish diverse and effective ongoing engagement channels, including bi-directional feedback and EHR-focused committees, led by facility leaders and supported by intermediaries, to enhance user involvement and system improvement.
	Address challenges in large organizations by streamlining communication, and provide periodic updates for major system changes

### Strengths and limitations

A notable strength of the study is that it builds on two existing reviews by Mair and Ross,^7,14^ which enhances their relevance and amplifies some of the findings from earlier reviews. However, the rapid review's limited search on PubMed and CINAHL only may have constrained our findings, potentially missing other information that may be captured by a more extensive search. The lead author (CZ), carried out the data extraction alone, however, the lack of cross checking at this stage was mitigated by supervisors, who checked for accuracy and consistency in the process and methodology.

## Conclusion

In conclusion, this study highlights the importance of user engagement for a successful EHR implementation. These strategies include a communication of policy and vision, preparation for implementation, training and education, on-demand technical support and ongoing user engagement. The strategies are effective at different stages of implementation and need to be aligned with user coherence, cognitive participation, collective action and reflexive monitoring.

## Supplemental Material

sj-docx-1-dhj-10.1177_20552076241291286 - Supplemental material for What engagement strategies are useful in facilitating the implementation of electronic health records in health care settings? A rapid review of qualitative evidence synthesis using the normalization process theory Supplemental material, sj-docx-1-dhj-10.1177_20552076241291286 for What engagement strategies are useful in facilitating the implementation of electronic health records in health care settings? A rapid review of qualitative evidence synthesis using the normalization process theory by Campion Zharima, Samantha Mhlanga, Saira Abdulla, Jane Goudge and Frances Griffiths in DIGITAL HEALTH

sj-xlsx-2-dhj-10.1177_20552076241291286 - Supplemental material for What engagement strategies are useful in facilitating the implementation of electronic health records in health care settings? A rapid review of qualitative evidence synthesis using the normalization process theory Supplemental material, sj-xlsx-2-dhj-10.1177_20552076241291286 for What engagement strategies are useful in facilitating the implementation of electronic health records in health care settings? A rapid review of qualitative evidence synthesis using the normalization process theory by Campion Zharima, Samantha Mhlanga, Saira Abdulla, Jane Goudge and Frances Griffiths in DIGITAL HEALTH

sj-xlsx-3-dhj-10.1177_20552076241291286 - Supplemental material for What engagement strategies are useful in facilitating the implementation of electronic health records in health care settings? A rapid review of qualitative evidence synthesis using the normalization process theory Supplemental material, sj-xlsx-3-dhj-10.1177_20552076241291286 for What engagement strategies are useful in facilitating the implementation of electronic health records in health care settings? A rapid review of qualitative evidence synthesis using the normalization process theory by Campion Zharima, Samantha Mhlanga, Saira Abdulla, Jane Goudge and Frances Griffiths in DIGITAL HEALTH

sj-docx-4-dhj-10.1177_20552076241291286 - Supplemental material for What engagement strategies are useful in facilitating the implementation of electronic health records in health care settings? A rapid review of qualitative evidence synthesis using the normalization process theory Supplemental material, sj-docx-4-dhj-10.1177_20552076241291286 for What engagement strategies are useful in facilitating the implementation of electronic health records in health care settings? A rapid review of qualitative evidence synthesis using the normalization process theory by Campion Zharima, Samantha Mhlanga, Saira Abdulla, Jane Goudge and Frances Griffiths in DIGITAL HEALTH

## References

[1] Ayatollahi H Mirani N Haghani H . Electronic health records: what are the most important barriers? Perspect Health Inf Manag 2014; 11(Fall). PMC4272437 25593569

[2] Evans RS . Electronic health records: then, now, and in the future. Yearb Med Inform 2016; 25: S48–S61. 10.15265/IYS-2016-s006 PMC5171496 27199197

[3] Zhang Z Wang B Ahmed F , et al. The five Ws for information visualization with application to healthcare informatics. IEEE Trans Vis Comput Graph 2013; 19: 1895–1910. 24029909 10.1109/TVCG.2013.89

[4] Horth RZ Wagstaff S Jeppson T , et al. Use of electronic health records from a statewide health information exchange to support public health surveillance of diabetes and hypertension. BMC Public Health 2019; 19: 1–7. 31412826 10.1186/s12889-019-7367-z PMC6694493

[5] Zharima C Griffiths F Goudge J . Exploring the barriers and facilitators to implementing electronic health records in a middle-income country: a qualitative study from South Africa. Front Digit Health [Internet] 2023; 5: 1207602. https://www.frontiersin.org/articles/10.3389/fdgth.2023.1207602/full . 37600481 10.3389/fdgth.2023.1207602 PMC10437058

[6] World Health Organization. Global strategy on digital health 2020–2025. Geneva: World Health Organization, 2021, Licence: CC BY-NC-SA 3.0 IGO.

[7] Ross J Stevenson F Lau R , et al. Factors that influence the implementation of e-health: a systematic review of systematic reviews (an update). Implement Sci 2016; 11: 1–12. 27782832 10.1186/s13012-016-0510-7 PMC5080780

[8] Stewart RE Williams N Byeon YV , et al. The clinician crowdsourcing challenge: using participatory design to seed implementation strategies. Implement Sci [Internet] 2019; 14: 63. https://implementationscience.biomedcentral.com/articles/10.1186/s13012-019-0914-2 . 31200730 10.1186/s13012-019-0914-2 PMC6570922

[9] Hartzler A McCarty CA Rasmussen LV , et al. Stakeholder engagement: a key component of integrating genomic information into electronic health records. Genet Med 2013; 15: 792–801. 24030437 10.1038/gim.2013.127 PMC3909653

[10] Sweeney K Griffiths F . Complexity and healthcare: an introduction. Oxon, UK: Radcliffe Medical Press Ltd.; 2002.

[11] Mishuris RG Palmisano J McCullagh L , et al. Using normalisation process theory to understand workflow implications of decision support implementation across diverse primary care settings. BMJ Health Care Inform 2019; 26(1). doi:10.1136/bmjhci-2019-100088 PMC7062348 31630113

[12] Klein TM Augustin M Kirsten N , et al. Attitudes towards using electronic health records of patients with psoriasis and dermatologists: a cross-sectional study. BMC Med Inform Decis Mak [Internet] 2020; 20: 344. https://bmcmedinformdecismak.biomedcentral.com/articles/10.1186/s12911-020-01302-y . 33380329 10.1186/s12911-020-01302-y PMC7772927

[13] Lorenzi NM Kouroubali A Detmer DE , et al. How to successfully select and implement electronic health records (EHR) in small ambulatory practice settings. BMC Med Inform Decis Mak [Internet] 2009; 9: 15. https://bmcmedinformdecismak.biomedcentral.com/articles/10.1186/1472-6947-9-15 . 19236705 10.1186/1472-6947-9-15 PMC2662829

[14] Mair FS May C O’Donnell C , et al. Factors that promote or inhibit the implementation of e-health systems: an explanatory systematic review. Bull World Health Organ 2012; 90: 357–364. 22589569 10.2471/BLT.11.099424 PMC3341692

[15] Schroeder D Luig T Finch TL , et al. Understanding implementation context and social processes through integrating normalization process theory (NPT) and the consolidated framework for implementation research (CFIR). Implement Sci Commun [Internet] 2022; 3: 13. https://implementationsciencecomms.biomedcentral.com/articles/10.1186/s43058-022-00264-8 . 35139915 10.1186/s43058-022-00264-8 PMC8826671

[16] Nguyen P Kohlbeck SA Levas M , et al. Implementation and initial analysis of Cardiff model data collection procedures in a level I trauma adult emergency department. BMJ Open 2022; 12: e052344. 10.1136/bmjopen-2021-052344 PMC8739060 34992109

[17] Fennelly O Cunningham C Grogan L , et al. Successfully implementing a national electronic health record: a rapid umbrella review. Int J Med Inf 2020; 144: 104281. 10.1016/j.ijmedinf.2020.104281 PMC7510429 33017724

[18] O’Donnell A Kaner E Shaw C , et al. Primary care physicians’ attitudes to the adoption of electronic medical records: a systematic review and evidence synthesis using the clinical adoption framework. BMC Med Inform Decis Mak [Internet] 2018; 18: 101. https://bmcmedinformdecismak.biomedcentral.com/articles/10.1186/s12911-018-0703-x . 30424758 10.1186/s12911-018-0703-x PMC6234586

[19] May CR Mair F Finch T , et al. Development of a theory of implementation and integration: normalization process theory. Implement Sci [Internet] 2009; 4: 29. http://implementationscience.biomedcentral.com/articles/10.1186/1748-5908-4-29 . 19460163 10.1186/1748-5908-4-29 PMC2693517

[20] Murray E Treweek S Pope C , et al. Normalisation process theory: a framework for developing, evaluating and implementing complex interventions. BMC Med [Internet] 2010; 8: 63. http://bmcmedicine.biomedcentral.com/articles/10.1186/1741-7015-8-63 . 20961442 10.1186/1741-7015-8-63 PMC2978112

[21] Carter H Beard D Harvey A , et al. Using normalisation process theory for intervention development, implementation and refinement in musculoskeletal and orthopaedic interventions: a qualitative systematic review. Implement Sci Commun [Internet] 2023; 4: 114. https://implementationsciencecomms.biomedcentral.com/articles/10.1186/s43058-023-00499-z . 37723546 10.1186/s43058-023-00499-z PMC10506319

[22] Huddlestone L Turner J Eborall H , et al. Application of normalisation process theory in understanding implementation processes in primary care settings in the UK: a systematic review. BMC Fam Pract [Internet] 2020; 21: 52. https://bmcfampract.biomedcentral.com/articles/10.1186/s12875-020-01107-y . 32178624 10.1186/s12875-020-01107-y PMC7075013

[23] Finch TL Mair FS O’Donnell C , et al. From theory to “measurement” in complex interventions: methodological lessons from the development of an e-health normalisation instrument. BMC Med Res Methodol [Internet] 2012; 12: 69. https://bmcmedresmethodol.biomedcentral.com/articles/10.1186/1471-2288-12-69 . 22594537 10.1186/1471-2288-12-69 PMC3473304

[24] Moons P Goossens E Thompson DR . Rapid reviews: the pros and cons of an accelerated review process. Eur J Cardiovasc Nurs 2021; 20: 515–519. 34007994 10.1093/eurjcn/zvab041

[25] Tong A Flemming K McInnes E , et al. Enhancing transparency in reporting the synthesis of qualitative research: ENTREQ. BMC Med Res Methodol 2012; 12: 1–8. 23185978 10.1186/1471-2288-12-181 PMC3552766

[26] O’Brien BC Harris IB Beckman TJ , et al. Standards for reporting qualitative research: a synthesis of recommendations. Acad Med 2014; 89: 1245–1251. 24979285 10.1097/ACM.0000000000000388

[27] Hanckel B Petticrew M Thomas J , et al. The use of qualitative comparative analysis (QCA) to address causality in complex systems: a systematic review of research on public health interventions. BMC Public Health 2021; 21: 77. 33962595 10.1186/s12889-021-10926-2 PMC8103124

[28] Craven CK Sievert MC Hicks LL , et al. Experts speak: advice from key informants to small, rural hospitals on implementing the electronic health record system. In: MEDINFO 2013. IOS Press; 2013, p.608–612. 23920628

[29] McAlearney AS Sieck C Hefner J , et al. Facilitating ambulatory electronic health record system implementation: evidence from a qualitative study. BioMed Res Int 2013;2013(1):629574. 10.1155/2013/629574 PMC3817798 24228257

[30] Schoville RR . Discovery of implementation factors that lead to technology adoption in long-term care. J Gerontol Nurs [Internet] 2017; 43: 21–26. https://journals.healio.com/doi/10.3928/00989134-20170914-06 . 10.3928/00989134-20170914-06 28945269

[31] Alzghaibi HA Hutchings HA . Exploring facilitators of the implementation of electronic health records in Saudi Arabia. BMC Med Inform Decis Mak 2022; 22: 21. 36476224 10.1186/s12911-022-02072-5 PMC9730584

[32] Ser G Robertson A Sheikh A . A qualitative exploration of workarounds related to the implementation of national electronic health records in early adopter mental health hospitals. Lovis C, editor. PLoS ONE [Internet] 2014; 9:e77669. https://dx.plos.org/10.1371/journal.pone.0077669 . 10.1371/journal.pone.0077669 PMC3894172 24454678

[33] Sheikh A Cornford T Barber N , et al. Implementation and adoption of nationwide electronic health records in secondary care in England: final qualitative results from prospective national evaluation in “early adopter” hospitals. BMJ [Internet] 2011; 343:d6054–d6054. https://www.bmj.com/lookup/doi/10.1136/bmj.d6054 . doi:10.1136/bmj.d6054 PMC3195310 22006942

[34] Cresswell K Morrison Z Crowe S , et al. Anything but engaged: user involvement in the context of a national electronic health record implementation. Inform Prim Care 2011 July 1; 19(4). 10.14236/jhi.v19i4.814 22828574

[35] Rozenblum R Jang Y Zimlichman E , et al. A qualitative study of Canada’s experience with the implementation of electronic health information technology. Can Med Assoc J [Internet] 2011; 183:E281–E288. http://www.cmaj.ca/cgi/doi/10.1503/cmaj.100856 . 10.1503/cmaj.100856 PMC3060213 21343262

[36] Scantlebury A Sheard L Watt I , et al. Exploring the implementation of an electronic record into a maternity unit: a qualitative study using normalisation process theory. BMC Med Inform Decis Mak [Internet] 2017; 17: 4. http://bmcmedinformdecismak.biomedcentral.com/articles/10.1186/s12911-016-0406-0 28061781 10.1186/s12911-016-0406-0 PMC5219748

[37] Topaz M Ronquillo C Peltonen LM , et al. Nurse informaticians report low satisfaction and multi-level concerns with electronic health records: results from an international survey. AMIA Annu Symp Proc AMIA Symp 2016; 2016: 2016–2025. 28269961 PMC5333337

[38] Sherer SA Meyerhoefer CD Sheinberg M , et al. Integrating commercial ambulatory electronic health records with hospital systems: an evolutionary process. Int J Med Inf [Internet] 2015; 84: 683–693. https://linkinghub.elsevier.com/retrieve/pii/S1386505615001033 . 10.1016/j.ijmedinf.2015.05.010 26045022

[39] Swanik S . Implementation of an EMR system for a comprehensive dental service within a large regional hospital network: challenges and opportunities presented by the introduction of new technology. Online J Public Health Inform 2019 Sep 20; 11(2): e62587. 10.5210/ojphi.v11i2.10131 PMC6788901 31632613

[40] McAlearney AS Hefner JL Sieck CJ , et al. The journey through grief: insights from a qualitative study of electronic health record implementation. Health Serv Res 2015; 50: 462–488. 25219627 10.1111/1475-6773.12227 PMC4369218

[41] Fernald DH Wearner R Dickinson WP . The journey of primary care practices to meaningful use: a Colorado beacon consortium study. J Am Board Fam Med 2013; 26: 603–611. 24004712 10.3122/jabfm.2013.05.120344

[42] Rantz MJ Alexander G Galambos C , et al. The use of bedside electronic medical record to improve quality of care in nursing facilities: a qualitative analysis. CIN Comput Inform Nurs [Internet] 2011; 29: 149–156. https://journals.lww.com/00024665-201103000-00004 . 20975545 10.1097/NCN.0b013e3181f9db79

[43] Cucciniello M Lapsley I Nasi G , et al. Understanding key factors affecting electronic medical record implementation: a sociotechnical approach. BMC Health Serv Res 2015; 15: 1–19. 26184405 10.1186/s12913-015-0928-7 PMC4504039

[44] Costa GMC de Andrade IM de M Celino SD , et al. Functioning of the citizen’s electronic medical records in the prison system. Cienc Saude Coletiva 2022; 27: 4381–4388. 10.1590/1413-812320222712.10442022 36383851

[45] Sittig DF Ash JS Wright A , et al. How can we partner with electronic health record vendors on the complex journey to safer health care? J Healthc Risk Manag 2020; 40: 34–43. 32648286 10.1002/jhrm.21434

[46] McCrorie C Benn J Johnson OA , et al. Staff expectations for the implementation of an electronic health record system: a qualitative study using normalisation process theory. BMC Med Inform Decis Mak 2019; 19: 1–14. 31727063 10.1186/s12911-019-0952-3 PMC6854727

[47] Gui X Chen Y Zhou X , et al. Physician champions’ perspectives and practices on electronic health records implementation: challenges and strategies. JAMIA Open 2020; 3: 53–61. 32607488 10.1093/jamiaopen/ooz051 PMC7309228

[48] Holden RJ . What stands in the way of technology-mediated patient safety improvements? A study of facilitators and barriers to physicians’ use of electronic health records. J Patient Saf 2011; 7: 193. 22064624 10.1097/PTS.0b013e3182388cfa PMC3220192

[49] Upadhyay S Fen HH . A qualitative analysis of the impact of electronic health records (EHR) on healthcare quality and safety: clinicians’ lived experiences. Health Serv Insights [Internet] 2022 Jan; 15: 117863292110707. http://journals.sagepub.com/doi/10.1177/11786329211070722. 10.1177/11786329211070722 PMC8902175 35273449

[50] Bristol AA Nibbelink CW Gephart SM , et al. Nurses’ use of positive deviance when encountering electronic health records-related unintended consequences. Nurs Admin Q 2018; 42: E1–E11. 10.1097/NAQ.0000000000000264 29194338

[51] Abramson EL Patel V Malhotra S , et al. Physician experiences transitioning between an older versus newer electronic health record for electronic prescribing. Int J Med Inf 2012; 81: 539–548. 10.1016/j.ijmedinf.2012.02.010 22465355

[52] Schnall R Gordon P Camhi E , et al. Perceptions of factors influencing use of an electronic record for case management of persons living with HIV. AIDS Care [Internet] 2011; 23: 357–365. https://www.tandfonline.com/doi/full/10.1080/09540121.2010.507745 . 21347899 10.1080/09540121.2010.507745 PMC3129034

[53] Mohammed-Rajput NA Smith DC Mamlin B , et al. OpenMRS, a global medical records system collaborative: factors influencing successful implementation. In: AMIA annual symposium proceedings. American Medical Informatics Association; 2011, p.960. PMC3243141 22195155

[54] Shiells K Diaz Baquero AA Štěpánková O , et al. Staff perspectives on the usability of electronic patient records for planning and delivering dementia care in nursing homes: a multiple case study. BMC Med Inform Decis Mak [Internet] 2020; 20: 159. https://bmcmedinformdecismak.biomedcentral.com/articles/10.1186/s12911-020-01160-8 . 32660474 10.1186/s12911-020-01160-8 PMC7359585

[55] Furlong KE . EHR learning—it’s about nursing, leadership and long-term commitments. Nurs Leadersh Tor Ont 2016; 28: 38–47. 27122089 10.12927/cjnl.2016.24560

[56] Halas G Singer A Styles C , et al. New conceptual model of EMR implementation in interprofessional academic family medicine clinics. Can Fam Physician 2015; 61: e232–e239. PMC4430072 26167563

[57] Sieck CJ Pearl N Bright TJ , et al. A qualitative study of physician perspectives on adaptation to electronic health records. BMC Med Inform Decis Mak 2020; 20: 1–8. 32039728 10.1186/s12911-020-1030-6 PMC7008538

[58] De Vliegher K Paquay L Vernieuwe S , et al. The experience of home nurses with an electronic nursing health record: home nurses’ experience with ENR. Int Nurs Rev [Internet] 2010; 57: 508–513. https://onlinelibrary.wiley.com/doi/10.1111/j.1466-7657.2010.00827.x . 21050204 10.1111/j.1466-7657.2010.00827.x

[59] Mishra V Liebovitz D Quinn M , et al. Factors that influence clinician experience with electronic health records. Perspect Health Inf Manag 2022; 19: 1f. PMC9013220 35440924

[60] Grabenbauer L Fraser R McClay J , et al. Adoption of electronic health records. Appl Clin Inform 2011; 2: 165–176. 23616868 10.4338/ACI-2011-01-RA-0003 PMC3631919

[61] de A Rocha Gomes P Farah BF Silva Rocha R , et al. Electronic citizen record: an instrument for nursing care. Rev Pesqui Cuid E Fundam 2019;11(5).

[62] Doyle RJ Wang N Anthony D , et al. Computers in the examination room and the electronic health record: physicians’ perceived impact on clinical encounters before and after full installation and implementation. Fam Pract 2012; 29: 601–608. 22379185 10.1093/fampra/cms015

[63] Terry AL Ryan BL McKay S , et al. Towards optimal electronic medical record use: perspectives of advanced users. Fam Pract [Internet] 2018; 35: 607–611. https://academic.oup.com/fampra/article/35/5/607/4850640 . 29444228 10.1093/fampra/cmy002

[64] Chipps E Tucker S Labardee R , et al. The impact of the electronic health record on moving new evidence-based nursing practices forward. Worldviews Evid Based Nurs 2020; 17: 136–143. 32233009 10.1111/wvn.12435

[65] Watson CH Masalonis A Arnold T , et al. Methods and lessons learned from a current state workflow assessment following transition to a new electronic health record system. Perspect Health Inf Manag 2023; 20: 1c. PMC10245086 37293479

[66] Narattharaksa K Speece M Newton C , et al. Key success factors behind electronic medical record adoption in Thailand. J Health Organ Manag [Internet] 2016; 30: 985–1008. https://www.emerald.com/insight/content/doi/10.1108/JHOM-10-2014-0180/full/html . 27681029 10.1108/JHOM-10-2014-0180

[67] Chang CP Lee TT Liu CH , et al. Nurses’ experiences of an initial and reimplemented electronic health record use. CIN Comput Inform Nurs 2016; 34: 183–190. 26886680 10.1097/CIN.0000000000000222

[68] Meigs SL Solomon M . Electronic health record use a bitter pill for many physicians. Perspect Health Inf Manag 2016; 13(Winter). PMC4739443 26903782

[69] Walle AD Shibabaw AA Tilahun KN , et al. Readiness to use electronic medical record systems and its associated factors among health care professionals in Ethiopia: a systematic review and meta-analysis. Inform Med Unlocked [Internet] 2023; 36: 101140. https://linkinghub.elsevier.com/retrieve/pii/S2352914822002775 .

[70] Afrizal SH Hidayanto AN Handayani PW , et al. Narrative review for exploring barriers to readiness of electronic health record implementation in primary health care. Healthc Inform Res [Internet] 2019; 25: 141. http://e-hir.org/journal/view.php?id=10.4258/hir.2019.25.3.141 31406606 10.4258/hir.2019.25.3.141 PMC6689507

[71] Zurynski Y Ellis LA Tong HL , et al. Implementation of electronic medical records in mental health settings: scoping review. JMIR Ment Health [Internet] 2021; 8:e30564. https://mental.jmir.org/2021/9/e30564 . 10.2196/30564 PMC8456340 34491208

[72] Aguirre RR Suarez O Fuentes M , et al. Electronic health record implementation: a review of resources and tools. Cureus [Internet] 2019. https://www.cureus.com/articles/21899-electronic-health-record-implementation-a-review-of-resources-and-tools . 10.7759/cureus.5649 PMC6822893 31700751

[73] Palacholla RS Fischer N Coleman A , , et al. Provider- and patient-related barriers to and facilitators of digital health technology adoption for hypertension management: scoping review. JMIR Cardio [Internet] 2019; 3:e11951. http://cardio.jmir.org/2019/1/e11951/ . 10.2196/11951 PMC6834226 31758771

[74] Whitelaw S Pellegrini DM Mamas MA , et al. Barriers and facilitators of the uptake of digital health technology in cardiovascular care: a systematic scoping review. Eur Heart J—Digit Health [Internet] 2021; 2: 62–74. https://academic.oup.com/ehjdh/article/2/1/62/6128570 . 34048508 10.1093/ehjdh/ztab005 PMC8139413

[75] Webster PC . The rise of open-source electronic health records. The Lancet [Internet] 2011; 377: 1641–1642. https://linkinghub.elsevier.com/retrieve/pii/S0140673611606594 . 10.1016/s0140-6736(11)60659-4 21591284

[76] Ferry AM Davis MJ Rumprecht E , et al. Medical documentation in low- and middle-income countries: lessons learned from implementing specialized charting software. Plast Reconstr Surg—Glob Open [Internet] 2021; 9:e3651. https://journals.lww.com/10.1097/GOX.0000000000003651 . 10.1097/GOX.0000000000003651 PMC8219254 34168942

[77] Ali SK Khan H Shah J , et al. An electronic health record system implementation in a resource limited country—lessons learned. Digit Health [Internet] 2023 Sep; 9: 20552076231203660. http://journals.sagepub.com/doi/10.1177/20552076231203660. 10.1177/20552076231203660 PMC10515600 37744747

[78] Robertson A Bates DW Sheikh A . The rise and fall of England’s national programme for IT. J R Soc Med 2011; 104: 434–435. London, UK: SAGE Publications Sage UK. 22048671 10.1258/jrsm.2011.11k039 PMC3206716

[79] Califf CB Sarker S Sarker S . The bright and dark sides of technostress: a mixed-methods study involving healthcare IT. MIS Q [Internet] 2020; 44: 809–856. https://misq.org/the-bright-and-dark-sides-of-technostress-a-mixed-methods-study-involving-healthcare-it.html .

[80] Macabasag RLA Mallari EU Pascual PJC , et al. Normalisation of electronic medical records in routine healthcare work amidst ongoing digitalisation of the Philippine health system. Soc Sci Med [Internet] 2022; 307: 115182. https://linkinghub.elsevier.com/retrieve/pii/S0277953622004889 . 35797835 10.1016/j.socscimed.2022.115182

[81] Alobayli F O’Connor S Holloway A , et al. Electronic health record stress and burnout among clinicians in hospital settings: a systematic review. Digit Health [Internet] 2023 Dec;9: 20552076231220241. http://journals.sagepub.com/doi/10.1177/20552076231220241. 10.1177/20552076231220241 PMC10734365 38130797

[82] Babbott S Manwell LB Brown R , et al. Electronic medical records and physician stress in primary care: results from the MEMO study. J Am Med Inform Assoc [Internet] 2014; 21: e100–e106. https://academic.oup.com/jamia/article-lookup/doi/10.1136/amiajnl-2013-001875. 10.1136/amiajnl-2013-001875 PMC3957395 24005796

[83] Agyeman-Manu K Ghebreyesus TA Maait M , et al. Prioritising the health and care workforce shortage: protect, invest, together. Lancet Glob Health 2023; 11: e1162–e1164. 10.1016/S2214-109X(23)00224-3 PMC10191605 37209702

[84] Rahal RM Mercer J Kuziemsky C , et al. Factors affecting the mature use of electronic medical records by primary care physicians: a systematic review. BMC Med Inform Decis Mak [Internet] 2021; 21: 67. https://bmcmedinformdecismak.biomedcentral.com/articles/10.1186/s12911-021-01434-9 . 33607986 10.1186/s12911-021-01434-9 PMC7893965

