# Supplementary material for: What engagement strategies are useful in facilitating the implementation of electronic health records in health care settings? A rapid review of qualitative evidence synthesis using the normalization process theory
Source: Digit Health. 2024 Nov 3;10:20552076241291286. doi: 10.1177/20552076241291286 (PMC11533323; doi:10.1177/20552076241291286)
Supplement: sj-docx-1-dhj-10.1177_20552076241291286 - Supplemental material for What engagement strategies are useful in facilitating the implementation of electronic health records in health care settings? A rapid review of qualitative evidence synthesis using the normalization process theory [file sj-docx-1-dhj-10.1177_20552076241291286.docx]

**Appendix A: Search strategies and terms for PubMed and CINAHL**

**Search 1: PubMed**

| Search strategy |
| --- |
| 1. “Electronic Health Record” [Mesh] 2. “Health Record, Electronic” 3. “Health Records, Electronic” 4. “Electronic Medical Records” 5. “Electronic Medical Record” 6. “Medical Record, Electronic” 7. “Medical Records, Electronic” 8. “Medical Records, Computerized” 9. “Medical Record, Computerized” 10. “Computerized Medical Record” 11. “Computerized Medical Records” 12. #1 or #2 or #3 or #4 or #5 or #6 or #7 or #8 or #9 or #10 or #11 13. "Routin"[title/abstract] 14. "Normali?*" [Title/abstract] 15. "Integrat*"[title/abstract] 16. "Facilitate"[title/abstract] 17. "Barrier"[title/abstract] 18. "Implement"[title/abstract] 19. "Adopt"[title/abstract] 20. #13 or #14 or #15 or #16 or #17 or #18 or #19 21. #12 and #20 22. "Qualitative" 23. #21 and #22 24. Filter 2010 – 2023 |

**Search 2: CINAHL**

| Search strategy |
| --- |
| 1. TX – “Electronic Health Record*” 2. TX – “Health Record, Electronic*” 3. TX – “Health Records, Electronic*” 4. TX – “Electronic Medical Records*” 5. TX – “Electronic Medical Record*” 6. TX – “Medical Record, Electronic*” 7. TX – “Medical Records, Electronic*” 8. TX – “Medical Records, Computerized*” 9. TX – “Medical Record, Computerized*” 10. TX – “Computerized Medical Record*” 11. TX – “Computerized Medical Records*” 12. TX - #1 or #2 or #3 or #4 or #5 or #6 or #7 or #8 or #9 or #10 or #11 or #12 13. AB - "Routin" 14. AB - "Normali?*" 15. AB - "Integrat*" 16. AB - "Facilitate" 17. AB - "Barrier" 18. AB - "Implement" 19. AB - "Adopt" 20. #13 or #14 or #15 or #16 or #17 or #18 or #19 21. #12 and #20 22. AB - "Qualitative" 23. #21 and #22 24. Filter dates 2010 – 2023 25. Filter English language |

Key:

AB – Abstract search (also includes title)

TX – Full text search
